# Gut Mucosa in HIV Infection: “Immune Milk” Solution

**DOI:** 10.1371/journal.pmed.0040085

**Published:** 2007-02-27

**Authors:** Shawn J Green

In recent weeks, three noteworthy papers, published in *PLoS Medicine*, The *Journal of Virology*, and *Nature Medicine*, direct our attention to the gut as a critical target in HIV-1 infection and portal for therapeutic intervention.

In *PLoS Medicine*, Mehandru and colleagues report that over half of the CD4^+^ T cells in the gut mucosa are lost within the first few weeks after HIV-1 infection and remain consistently low, compared to peripheral blood sources, despite long term antiretroviral therapy; furthermore, of the few CD4^+^ T cells that persist in the gut, a significant increase in immune activation is observed [1]. Consistent with earlier observation in SIV models, Veazey reminds us that the battle against HIV-1 should focus on the intestinal mucosa with therapeutic strategies to reduce gut immune activation [2].

The longitudinal study in The *Journal of Virology* by Guadalupe and colleagues showed a similar discordance in CD4^+^ T cells between restoration in peripheral blood and significant delay in the gut mucosa of chronic infected individuals during antiretroviral therapy. Here, the depletion in CD4^+^ T cells was associated with an increase in gut immune activation, CD8^+^ T cells, and associated inflammation with a corresponding decease in epithelial growth and repair-associated genes in gut mucosal tissue [3]. Brenchley and colleagues suggest in *Nature Medicine* that HIV infection causes this breakdown in the gut mucosa resulting in a “leaky gut,” thus allowing translocation of gut-derived endotoxin and the subsequent triggering of immune activation [4].

Collectively, these observations suggest that an orally active therapeutic, used in conjunction with antiretroviral therapy, be designed to both block gut-derived microbial translocation and stimulate restitution of the gut epithelium. The hope would be to restore immunological integrity of the intestinal mucosal barrier, thereby controlling immune activation, both locally, in the gut mucosa, and systemically by suppressing cellular targets distal to the gut that may directly contribute to the progression of AIDS [5–8].

The design for such an orally active therapeutic may be found in the complex formula of bovine colostrum and “immune milk,” which has long been recognized to offer passive protection from a broad number of enteric bacterial and viral pathogens, primarily via the transfer of immunoglobulins and suppression of gut-associated inflammation with promotion of mucosal repair and regeneration.

The gut in chronic HIV-1-infected individuals appears to be reminiscent of newborn calves. Calves are born with a highly immature mucosal immune system and “leaky gut” which, if not immediately corrected, results in death due to infection and associated systemic immune activation. However, the cow's first milk rescues her calves from harmful gut microbes with a uniquely complex cocktail enriched with neutralizing polyclonal antibodies, cytokine tissue repair factors, and immune enhancing probiotics, such as *Lactobacillus* species.

Regular consumption of biologically active bovine colostrum has been known for years to promote the development of infantile gut-associated lymphoid tissue and enhance CD4^+^ levels, while suppressing CD8^+^ and inflammatory bowl disease (IBD), including ulcerative colitis and Crohn disease [5]. The severity of IBD is often correlated with gut microbial-endotoxin translocation, which now appears in chronic HIV-1-infected individuals [4]. Similar to bovine colostrum, “immune milk” from properly vaccinated cows affords passive immunity against bacterial, viral, and fungal infections in the human gastrointestinal tract, as well as taming gut inflammation [8].

Hence, there may be lessons learned from Bessie's “immune milk.” If viewed as a unique formula that has evolved to complement gut immunity, “immune milk” may also provide relief in chronic HIV-infected individuals. Initial studies have already shown that ingestion of colostrum alleviates refractory diarrhea in HIV patients with a corresponding increase in both body weight and peripheral blood CD4^+^ T cells [9,10]. As we learn more about the gut microenvironment in HIV-infected individuals, Bessie may prove to be a worthwhile platform for the consideration of “immune milk” exhibiting both microbial endotoxin and HIV-neutralizing activity along with its innate anti-inflammatory and tissue repair and regenerative properties.
